# Postoperative adjuvant chemotherapy in patients with stage II early onset colorectal cancer: exploration and discovery using real-world data and the SEER database

**DOI:** 10.3389/fonc.2025.1566569

**Published:** 2025-05-30

**Authors:** Shikun Cao, Yihuan Qiao, Lijun Wu, Qi Chen, Kai Tian, Jipeng Li, Chungen Xing

**Affiliations:** ^1^ Department of General Surgery, The Second Affiliated Hospital of Soochow University, Suzhou Jiangsu, China; ^2^ Department of Digestive Surgery, The First Affiliated Hospital of Air Force Medical University, Xi’an, Shaanxi, China

**Keywords:** early onset colorectal cancer, adjuvant chemotherapy, deficient mismatch repair, survival, landmark analysis

## Abstract

**Background and aim:**

The incidence of early-onset colorectal cancer (EOCRC) is rising, yet intensive postoperative adjuvant chemotherapy (ACT) often results in overtreatment with minimal prognostic benefit. This study aims to assess the therapeutic necessity of ACT in stage II EOCRC patients and to identify potential ACT candidates.

**Methods:**

A total of 296 non-ACT and 50 ACT patients with stage II EOCRC were included from Xijing Hospital (XJCRC), and 2067 non-ACT and 1163 ACT patients were enrolled from the Surveillance, Epidemiology, and End Results (SEER) cohort. To address selection bias and confounding, propensity score matching, inverse probability treatment weighting (IPTW), and multivariate Cox regression analyses were utilized. Survival curves and landmark analysis were employed to compare Overall Survival (OS) differences.

**Results:**

Similar OS were observed between ACT and non-ACT groups in both cohorts before and after adjustment for confounders. No significant survival differences were noted in dMMR (P = 0.48), pMMR (P = 0.07), and T3 (P = 0.83) subgroups. However, T4 stage patients receiving ACT demonstrated prolonged survival compared to non-ACT counterparts, particularly after three years (P = 0.007), as identified by landmark analysis.

**Conclusions:**

Most stage II EOCRC patients might yield limited benefits from postoperative ACT, with the sole exception of those at T4 stage, who could experience long-term clinical advantages.

**Clinical Trial Registration:**

https://clinicaltrials.gov/study/, identifier NCT06308354.

## Introduction

Colorectal cancer (CRC) is the third leading cancer diagnosed globally and causes 900,000 cancer-related mortality per year (1). Notably, the incidence of early-onset colorectal cancer (EOCRC) is alarmingly increasing. This trend may be associated with adverse lifestyle factors, including poor diet, obesity, and lack of physical activity (2–4).

EOCRC is a highly heterogeneous disease and strikingly differs from the late onset CRC (LOCRC) among the clinical, pathological, and molecular characteristics. Specifically, EOCRC patients were characterized by more advanced T stages, higher prevalence of left-sided carcinoma, adverse histopathological features (poorly differentiated grade, perineural invasion, venous invasion, and mucinous and/or signet cell morphology), higher proportion of microsatellite instability-high (MSI-H)/deficient Mismatch Repair (dMMR) status and more germline mutations (5–8). At the same time, EOCRC patients with advanced stages were more likely to accept intensive therapies than old patients (9, 10). However, some studies demonstrated that unsatisfactory survival outcomes for patients with EOCRC were still seen as compared patients with LOCRC (11, 12). Meanwhile some other researchers unraveled opposite results that EOCRC had superior or comparable prognosis than their older counterparts (7, 9, 13–15).

Given this contradictory results, optimal treatment for EOCRC should be further explored and validated. Many studies have demonstrated that a significant proportion of EOCRC patients are overtreated and receive unnecessary treatments with potential long-term toxicity of chemotherapy (2, 10). A recent study found only minimal survival gain among EOCRC patients who underwent chemotherapy compared to LOCRC patients receiving fewer treatments (9). Therefore, the appropriate use of chemotherapy in the treatment of EOCRC warrants further investigation and evaluation. However, individualized therapeutic regimens for EOCRC patients are not well-established, with survival data lacking and conflicting.

It is widely accepted that stage II CRC patients with MSI-H/dMMR do not benefit from adjuvant chemotherapy (ACT) after surgery, especially from fluorouracil-based chemotherapy (16). Given the considerable prevalence of MSI-H/dMMR among EOCRC patients, it is clinically important to investigate the therapeutic impacts of postoperative ACT in stage II EOCRC patients (2). Patients with advanced T4 stage, who are stratified into high-risk stage II, are often considered optimal candidates for ACT. However, whether dMMR or T4 stage EOCRC patients are potential candidates for ACT remains unclear.

Therefore, our study aims to explore the necessity of ACT for stage II EOCRC patients and to identify the potential candidates for postoperative ACT.

## Methods

### Patients and study design

This retrospective cohort study of EOCRC was conducted utilizing data from Xijing Hospital in Shaanxi Province, China (XJCRC), and the SEER cohort (https://seer.cancer.gov). To explore whether Stage II EOCRC patients could benefit from the ACT regimens, we enrolled patients in the XJCRC cohort between December 2013 and December 2022, and in the SEER cohort with detailed clinical features between 2010 and 2015. In accordance with the Chinese Society of Clinical Oncology (CSCO) guidelines, the ACT regimens administered to eligible patients included monotherapy with fluorouracil-based regimens or the CAPEOX regimen. The EOCRC patients are defined as younger than 50 (< 50) when diagnosed (17). The inclusion criteria of our study were shown as followed: (1) diagnosis of EOCRC; (2) TNM II stage; (3) underwent radical operation; (4) complete detailed clinicopathological features and follow-up duration and (5) standard chemotherapy regimens if they were adopted ACT. The exclusion criteria were revealed as followed: (1) multiple primary tumors; (2) known hereditary syndrome; (3) younger than 18 years old; (4) less than one month of follow-up; (5) unavailable information about ACT; (6) incomplete clinical and histological factors such as retrieved lymph nodes (rLNs), MSI or MMR, tumor biomarkers, and tumor size; (7) preoperative or postoperative radiation, immunotherapies, and targeted therapies; (8) accompanied with preoperative intestinal obstruction or perforation and (9) resection margins positive or less than 2cm of circumference resection margin, bowel margin or anal margin.

In our investigation, stage II EOCRC patients were divided into two distinct cohorts: the ACT group, which underwent ACT postoperatively, and the non-ACT group, which was relegated to observation after surgery. To ameliorate the potential influence of confounders on the Overall Survival (OS) of stage II CRC or therapy allocation modalities, we employed Propensity Score Matching (PSM) and multivariate Cox regression analyses. This study was approved by the Medical Ethics Committee of Xijing Hospital of Air Force Medical University (No. KY20232232-F-1) in 2023. The register ID of Clinical Trial is NCT06308354.

### Clinical features and follow-up

Clinical variables, including sex, age, height, weight, body mass index (BMI), serum albumin, TNM stage, tumor size, ACT, and MMR/MSI status, were retrieved from electronic medical records, as previously described (18, 19). Levels of serum tumor biomarkers, such as carcinoembryonic antigen (CEA), cancer antigen 125 (CA125), and carbohydrate antigen 19-9 (CA199), were measured using a Cobas 8000 Analyzer (Roche Diagnostics, Mannheim, Germany). Postoperative pathological reports were reviewed to obtain data on the status of retrieved lymph nodes (rLNs), lymphatic vessel invasion (assessed with D2-40), perineural invasion (assessed with S100), and microvascular invasion (assessed with CD34).

For the SEER cohort, pathological tumor stage, tumor size, and tumor grade were classified according to the 7th edition of the American Joint Committee on Cancer (AJCC) TNM staging system. Additional clinical characteristics, such as demographic data and the number of rLNs, were also collected. With regard to ACT, patients with high-risk stage II disease (20) were administered standard chemotherapy regimens.

The primary endpoint was OS, which was defined as death associated with any cause and was calculated from diagnosis to last contact or mortality. The OS of the SEER cohort was defined using the SEER Vital status recode and survival time in the SEER registry (21).

### PSM and inverse probability treatment weighting

To address potential confounding and selection bias in our observational study, we employed a combination of propensity score matching (PSM), inverse probability of treatment weighting (IPTW), and multivariate Cox regression. PSM was used to create comparable groups by matching subjects based on their propensity scores, thereby balancing measured covariates between treatment groups and emulating a randomized controlled trial. However, PSM can be limited by sample size reduction and incomplete covariate balance. To overcome these limitations, IPTW was applied to weight subjects based on the inverse probability of receiving the treatment they actually received, ensuring the inclusion of all subjects and providing a more robust adjustment for covariates. Multivariate Cox regression was subsequently used to model time-to-event data and estimate hazard ratios for primary outcomes, allowing for further adjustment of additional covariates. This combined approach enhanced the robustness and reliability of our findings by addressing potential confounding and selection bias while maximizing statistical power and accuracy.

The PSM method was used to reduce the effect of selection bias and adjust for potential confounding factors (22). Propensity scores were derived by fitting a logistic regression model based on age, sex, BMI, CEA, CA125, CA199 levels, rLNs, MMR status and T stage for the XJCRC cohort while based on age, sex, tumor grade, tumor size, rLNs and T stage for the SEER cohort. The two groups were matched with a caliper width of 0.02, and a ratio of 1:1 nearest neighbors matching without replacement was performed with the ‘MatchIt’ package in R software.

For IPTW, stabilized weights were computed using the propensity score by multiplying the weight by the probability of the patients receiving the ACT administered. The distribution of covariates was then assessed after the application of these weights.

### Statistical analysis

All statistics were conducted in R software (version 4.12). The Kaplan-Meier (K-M) method was applied to estimate the survival distribution, which were tested by the log-rank test. Additionally, in crossing survival curves, we performed the landmark analysis with R package ‘jskm’ for all endpoints by dividing the entire follow-up period into the first three years and subsequent years (23). Hazard ratio (HR) and 95% confidence interval (CI) were calculated to evaluate the differences of OS. a restrictive cubic spline (RCS) function was applied to explore linear or nonlinear prognostic profiles of age in the XJCRC and SEER cohorts. A two-sided P < 0.05 was considered statistically significant.

## Results

### Basic clinical characteristics of II stage EOCRC patients

**Figure 1:**
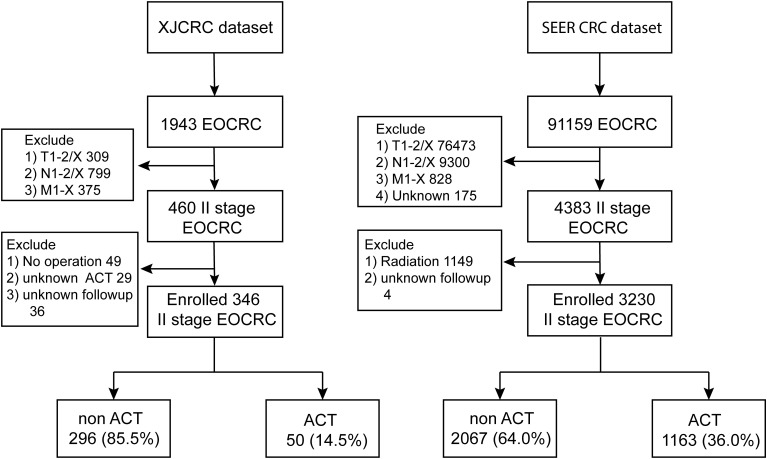
Flow chart of the study.

In the XJCRC cohort, a total of 1,943 patients diagnosed with EOCRC. After applying exclusion criteria to eliminate patients with T1-2/X (309 patients), N1-2/X (799 patients), and M1-X (375 patients) disease stages, a cohort of 460 patients with stage II EOCRC was obtained. Further, excluding 49 patients who did not undergo surgical intervention, 29 patients with unknown ACT regimens, and 36 patients with unavailable follow-up information, resulting in a final cohort of 346 patients with stage II EOCRC. Subsequently, these patients were stratified into two groups based on their treatment with ACT: 296 patients (85.5%) were categorized into the non-ACT group, and 50 patients (14.5%) were categorized into the ACT group (**Figure 1**). The 1-, 3- and 5- year of OS rate of II stage EOCRC was 99.1%, 97.1% and 94.1%, respectively (**Supplementary Figure 1**). The median age at diagnosis for the entire patient cohort was 43 years (IQR: 38.0–47.0 years). The median number of rLNs after surgery was 18[IQR:16.0;22.0].The overall prevalence of dMMR in the study population was 28%(**Table 1**). while, a significant difference was observed between the ACT and non-ACT groups, with the ACT group exhibiting a lower median number of rLNs compared to the non-ACT group (16 vs. 19, P = 0.005). Regarding the prevalence of dMMR, no significant difference was found between the ACT and non-ACT groups, with prevalence rates of 28.7% and 24.0%, respectively (P = 0.605; **Supplementary Table 1**).

**Table 1:** Basic characteristics of the II stage EOCRC in the XJCRC and SEER data cohort.

Characteristics	XJCRC	SEER
	N=346	N=3230
Gender		
Female	141 (40.8%)	1529 (47.3%)
Male	205 (59.2%)	1701 (52.7%)
Age	43.0 [38.0;47.0]	44.0 [40.0;47.0]
BMI	22.7 [20.0;25.2]	NA
Retrieved lymph node	18.0 [16.0;22.0]	21.0 [15.0;30.0]
S100		NA
Negative	277 (80.1%)	
Positive	169 (19.9%)	
CD34		NA
Negative	261 (75.4%)	
Positive	85 (24.6%)	
D240		NA
Negative	264 (76.3%)	
Positive	82 (23.7%)	
Grade		
Well differentiated	25 (7.2%)	261 (8.1%)
Moderately differentiated	239 (69.1%)	2362 (73.1%)
Poorly differentiated	56 (16.2%)	405 (12.5%)
Undifferentiated	0 (0%)	106 (3.3%)
Unknown	12 (2.5%)	96 (3.0%)
T stage		
T3	299 (86.4%)	2665 (82.5%)
T4	47 (13.6%)	565 (17.5%)
ACT		
No	296 (85.5%)	2067 (64.0%)
Yes	50 (14.5%)	1163 (36.0%)
MMR status		NA
dMMR	97 (28.0%)	
pMMR	249 (72.0%)	

XJCRC, Xijing hospital cohort; SEER, Surveillance, Epidemiology, and End Results; ACT, adjuvant chemotherapy; BMI, body mass index; MMR, Mismatch Repair.

In the SEER cohort, a total of 91159 patients diagnosed with EOCRC participated. After excluding 76,473 patients with T1-2/X, 9,300 patients with N1-2/X, 828 patients with M1-X, and 175 patients with unknown stage, 4383 patients with stage II EOCRC were obtained. Further, excluding 1,149 patients receiving radiation therapy and 4 patients with unknown follow-up data, and 3230 patients remained eligible for analyses, including 2067 non-ACT or observation patients (64.0%) and 1163 patients underwent ACT (36%; **Figure 1**). The 1-, 3- and 5- year of OS rate of SEER cohort EOCRC population was 97.9%, 92.0% and 87.7%, respectively (**Supplementary Figure 2**). The median age at diagnosis was 44 years while the patients receiving ACT were observed to be younger than the non-ACT population (44 vs 45, P < 0.001, **Table 1**; **Supplementary Table 2**). The median count of rLNs was 21, far more than the standard 12 as the least requirement. As expected, more ratio of well-differentiated, modern-differentiated tumor grade and T3 stage (89.8% vs 69.6%) were observed in the non-ACT group compared with the ACT group (**Supplementary Table 2**).

**Figure 2:**
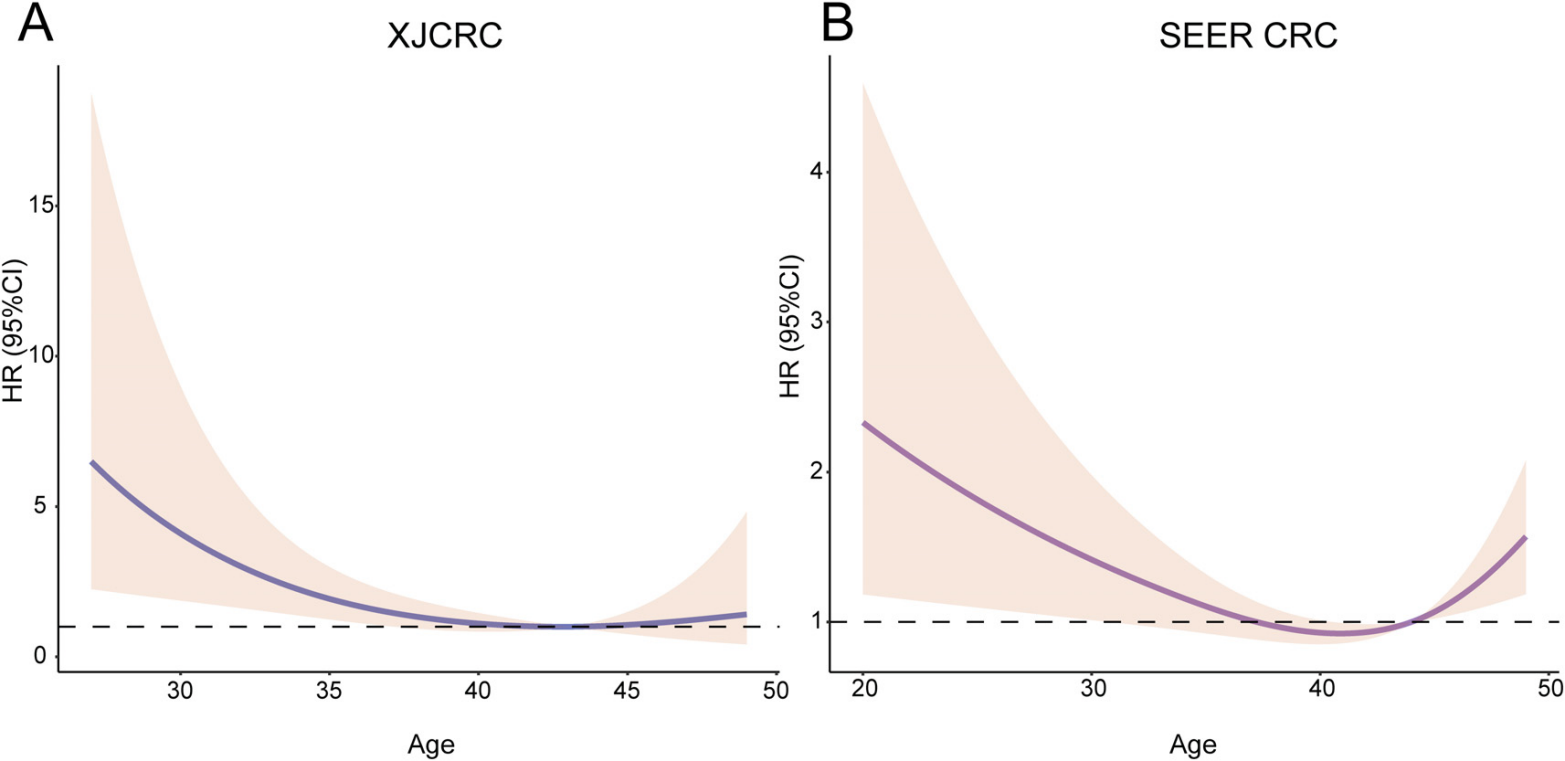
RCS of age and HR among the EOCRC patients. Relationship between age and HR among the XJCRC **(A)** and SEER EOCRC patients **(B)** identified by RCS curves. RCS, restrictive cubic spline; HR, hazard ratio; XJCRC, Xijing hospital CRC cohort; EOCRC, early onset colorectal cancer; SEER, Surveillance, Epidemiology, and End Results.

Subsequently, an RCS function was utilized to delineate the potential linear or nonlinear associations between age and survival outcomes within the XJCRC and SEER cohorts. RCS curves showed that there was a linear relationship between age and OS in the XJCRC cohort (P = 0.1473; **Figure 2A**) while a nonlinear correlation in the SEER cohort (P = 0.0009; **Figure 2B**).

### Prognostic implications of ACT on the overall populations of II stage

**Figure 3:**
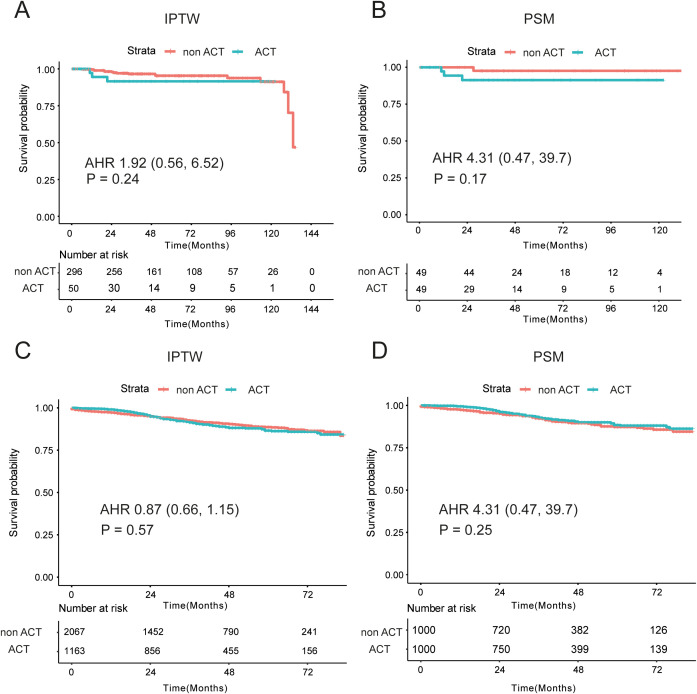
Prognostic differences between non-ACT and ACT groups in the XJCRC and SEER cohort. **(A, B)** Prognostic differences of two groups in the XJCRC cohort, by the IPTW **(A)** and PSM **(B)** adjusted; **(C, D)** Prognostic differences of two groups in the SEER cohort, by the IPTW **(C)** and PSM **(D)** adjusted; ACT, adjuvant chemotherapy; PSM, Propensity Score Matching; IPTW, inverse probability treatment weighting; AHR, adjusted hazard ratio.

To investigate whether II stage EOCRC could receive benefit from the ACT, firstly, we compared the OS of ACT and non-ACT group in the whole EOCRC population. By the IPTW analysis, similar OS was observed in the ACT and non-ACT groups in the XJCRC cohort (adjusted HR = 1.92; P = 0.24; **Figure 3A**). There were no significant disparities of high-risk factor, including S100, CD34 and D240 markers, between ACT and non-ACT groups (**Supplementary Table 1**). Meanwhile, the non-ACT patients possessed more rLNs than ACT group. It was reported that more lymph nodes were harvested during the operation, the OS of patients were better (24–26). Therefore, we reduced the confounding effects of rLNs by the PSM method. After the PSM, there were no noticeable differences of variables in two groups (all P > 0.05; **Supplementary Table 1**) and the analogous survival curves were still found in the survival curve (P = 0.17, **Figure 3B**).

In the SEER cohort, a highly overlapping survival curves were observed in the ACT and non-ACT groups, identified by the IPTW method (adjusted HR = 0.87, P = 0.57, **Figure 3C**). However, there were some apparent differences of clinical factors in the two groups, where non-ACT patients were characterized by older, more ratio of male, well- and moderate- differentiated tumor grade (83.2% vs 77.7%), T3 (89.8% vs 69.6%) and smaller tumor size than ACT patients (**Supplementary Table 2**). Then, PSM analysis was conducted to mitigate the confounding effects of these unevenly distributed variables, and there were no significant differences of age, sex, tumor grade, T stage and tumor size in the two groups (**Supplementary Table 2**). Comparable oncological outcomes were still identified in the ACT and non-ACT groups (P = 0.25, **Figure 3D**).

Apart from the PSM method and survival curves, we also implemented the multivariate Cox analysis to adjust some well-established confounders. In the XJCRC cohort, ACT could not be an independent factor for the OS of II stage patients before (P = 0.235) and after PSM (P = 0.578; **Table 2**). In the SEER cohort, T stage and tumor grade were independent prognostic clinical factors before the PSM while T stage were remained for OS factors after the PSM. Consistent with the result of XJCRC cohort, the ACT was still not an independent prognostic factor in the SEER cohort before (P = 0.134) and after implementation of PSM method (P = 0.196; **Table 2**).

**Table 2:** Multivariate Cox analysis of the II stage EOCRC in the XJCRC and SEER cohort.

Population	Characteristics	Before PSM			After PSM		
		HR	95% CI	P value	HR	95% CI	P value
XJCRC cohort	ACT			0.235			0.578
	No	—	—		—	—	
	Yes	2.35	0.64, 8.66		1.96	0.17, 22.6	
	T stage			0.374			0.525
	T3	—	—		—	—	
	T4	1.91	0.49, 7.37		0.00	0.00, 14.23	
	BMI	1.00	0.87, 1.14	0.965	1.00	0.71, 1.40	0.992
	MMR status			0.590			0.112
	dMMR	—	—		—	—	
	pMMR	1.32	0.48, 3.64		42.68	0.00, 98.11	
SEER cohort	ACT			0.134			0.196
	No	—	—		—	—	
	Yes	0.82	0.64, 1.06		0.82	0.60, 1.11	
	T stage			<0.001			<0.001
	T3	—	—		—	—	
	T4	2.02	1.44, 2.82		1.93	1.26, 2.95	
	Grade			<0.001			0.221
	Moderately differentiated	—	—		—	—	
	Well differentiated	1.00	0.63, 1.58		0.67	0.32, 1.38	
	Poorly differentiated	1.61	1.18, 2.19		1.44	0.97, 2.12	
	Undifferentiated	2.00	1.21, 3.30		1.16	0.54, 2.50	
	Unknown	0.37	0.12, 1.16		0.68	0.21, 2.15	

BMI, body mass index; HR, Hazard Ratio; CI, Confidence Interval; ACT, adjuvant chemotherapy; XJCRC, Xijing hospital CRC cohort; SEER, Surveillance, Epidemiology, and End Results.

### Exploring the therapeutic impacts of ACT on subgroups of II stage EOCRC

Then, we explored the therapeutic impacts of ACT on the dMMR/proficient mismatch repair (pMMR), T3/4 stage patients by the subgroup analyses. In the dMMR subgroup, 85 patients underwent observation while only 12 patients were adopted ACT therapy. There were not obviously differences in the non-ACT and ACT groups (P > 0.05; **Supplementary Table 3**). By the IPTW analysis, patients with dMMR could not achieve clinical benefits from the ACT, as shown in the **Supplementary Figure 3A** (P = 0.48). After the PSM, comparably similar survival curves were observed in the non-ACT and ACT groups (P = 1, **Supplementary Figure 3B**). In the pMMR subgroup, 211 patients were adopted non-ACT while 38 patients underwent ACT (**Supplementary Table 4**). Survival analysis demonstrated patients with non-ACT had not significantly different OS than those with ACT (P = 0.07; **Supplementary Figure 3C**). After the PSM, the rLNs distributed equally in the ACT and non-ACT groups, and no statistical prognostic discrepancies were observed in the two groups (P = 0.33; **Supplementary Figure 3D**).

**Figure 4:**
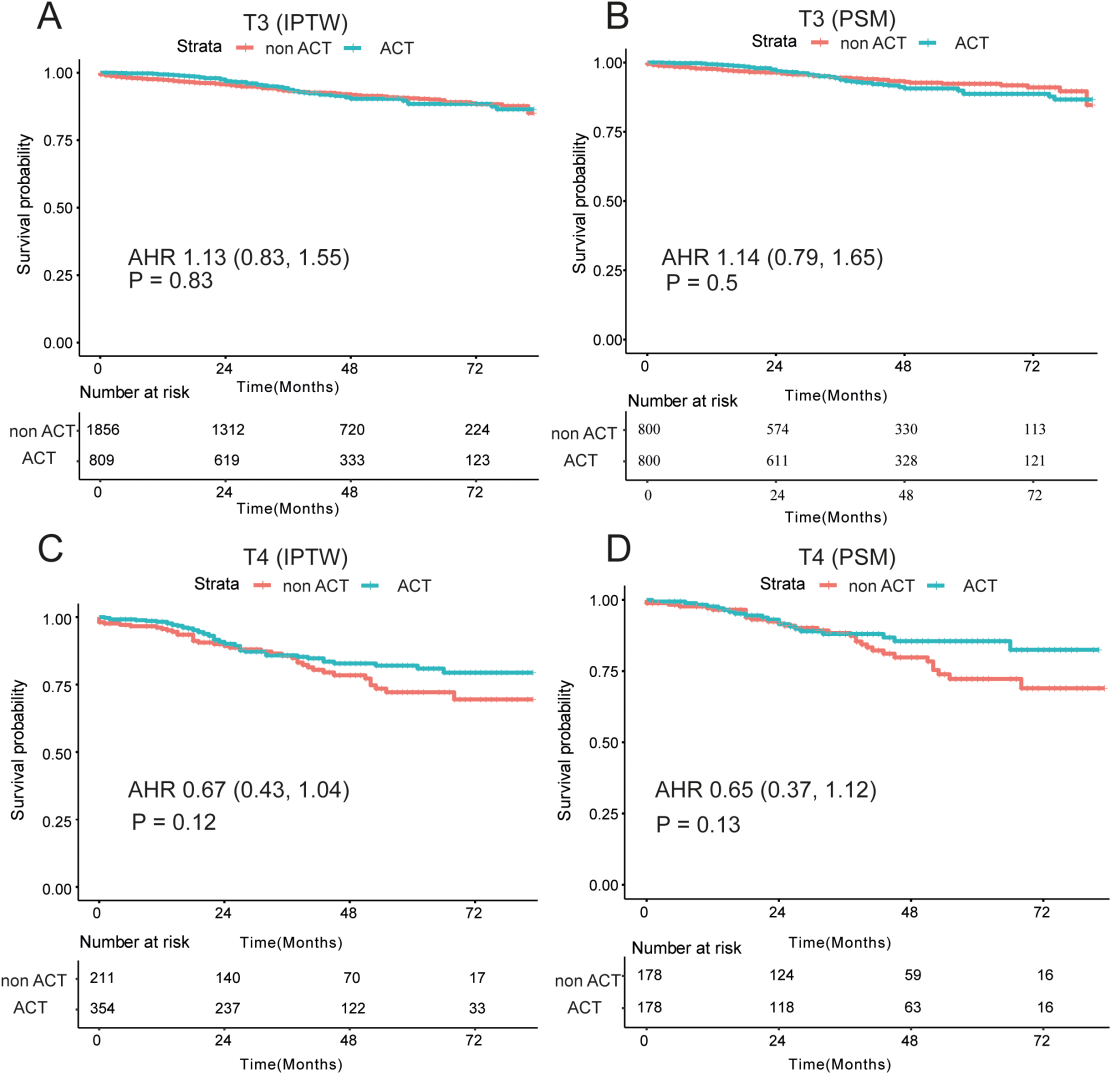
Survival differences among T3/T4 subgroups by IPTW and PSM analysis. **(A)** No significant differences of OS among the non-ACT and ACT groups with dMMR and pMMR patients by the IPTW **(A)** and the PSM **(B, C)** Prognostic differences among the non-ACT and ACT groups patients with T3 stage and T4 stage by the IPTW **(C)** and the PSM **(D)**; OS, overall survival; ACT, adjuvant chemotherapy; PSM, Propensity Score Matching; IPTW, inverse probability treatment weighting; AHR, adjusted hazard ratio.

**Figure 5:**
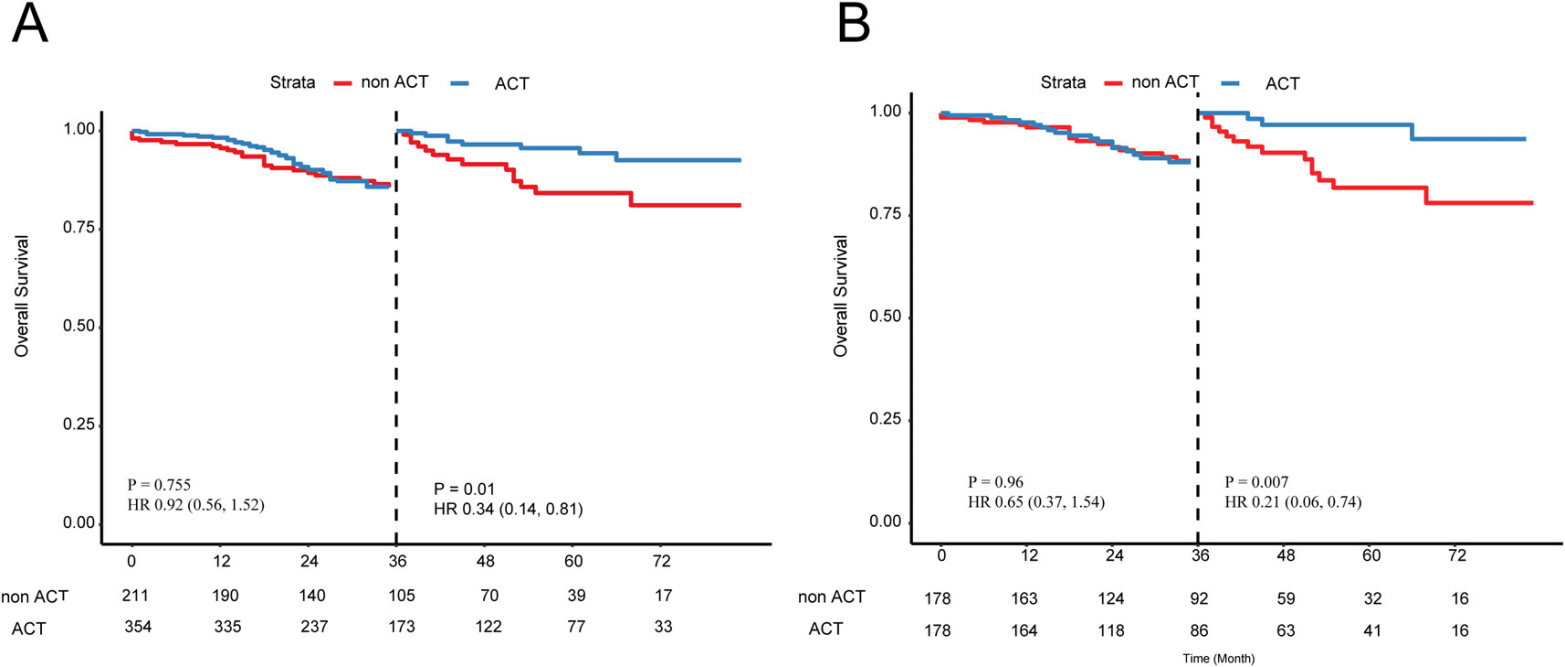
Landmark analysis of patients with T4 stage EOCRC **(A)** before and **(B)** after the PSM. PSM, Propensity Score Matching.

In the SEER cohort, among the T3 stage cohort, striking resemblances of survival curves between the ACT and non-ACT groups through the IPTW and survival analyses, demonstrating that T3 stage EOCRC patients could not yield clinical nets from the ACT (adjusted HR = 1.13; P = 0.83; **Figure 4A**). After adjustment of tumor grade by the PSM (**Supplementary Table 5**), the same results were revealed in the form of K-M curves (P = 0.5; **Figure 4B**). As for the T4 subgroup, 565 EOCRC patients were stratified into ACT (N = 354) and non-ACT (N = 211) groups. Among the basic clinical characteristics, the ACT group had more lymph nodes yield (22 vs 19, P = 0.028) while larger proportion of poorly differentiated (19.5% vs 10.4%) and undifferentiated (5.9% vs 3.8%) tumor grade (**Supplementary Table 6**). The survival curves demonstrated that there were no distinctly prognostic discrepancies in the ACT and non-ACT groups (P = 0.12, **Figure 4C**). After the adjustment of tumor grade and rLNs, all basic clinical parameters were balanced between two groups. The K-M curves unraveled no significant differences (P = 0.13, **Figure 4D**). Intriguingly, we found that survival curves were highly matched in the first three years while the two K-M curves were noticeably separate in the latter years. Consequently, we conducted the landmark analysis to validate our findings, and as expected, in the first 36 months, the survival curves were highly similar (P = 0.961) while these patients with ACT had favorable outcomes than that without ACT after three years (P = 0.007; **Figure 5**).

## Discussion

Patients younger than 50 years at diagnosis are generally considered EOCRC (27). This demographic often retains reproductive aspirations. However, it is well-documented that oncological treatments can lead to iatrogenic infertility (17, 28, 29). Consequently, it is imperative to investigate the optimal candidate selection for ACT in stage II EOCRC patients to mitigate the risk of redundant therapy and preserve reproductive potential where possible. Here, we retrospectively incorporated two independent cohorts, namely XJCRC and SEER cohort, which represented eastern and western EOCRC populations. We found that overall EOCRC population with II stage could not benefit from the ACT. By the subgroup analyses, T4 stage patients underwent ACT had long-term survival benefits than those with non-ACT, especially three years after surgery.

The median age of II stage EOCRC patients was 43 and 44 years among the two cohorts, consistent with the previous reports that 75% young onset CRC patients arise in people 40 and <50 years of age (30, 31). Therefore, the high-risk CRC screening should be updated timely, and some countries endorsed that the screening time begin at 44 (in Italy), at 40 (in Australia) and 45 (in the USA) (4, 32, 33). The proportion of dMMR in our cohort was 28%, similar to the reported 26.2% of I-III stage of EOCRC patients (34). In addition, ratio of patients with dMMR allocated to ACT treatments was same as the pMMR populations (34), similar to our findings as well. Besides, we found that patients with dMMR/pMMR could not benefit from the ACT after surgery, and a recent study demonstrated that II stage patients with dMMR should adopt the immunotherapy rather than chemotherapy, even among T4 stage (35). In conclusion, dMMR or pMMR could not become an effective biomarker for adopting ACT, might be an indicator for immunotherapy, instead.

As for the T stage, we found that more than 80% patients were T3 stage while less than 20% were T4 stage. As we know, T4 stage is a well-established clinical indicator for postoperative combined chemotherapy while patients with T3 stage could accept observation or single-agent chemotherapy (36). However, different studies differ in the statistical methods and sample characteristics when assessing the benefit from ACT. Furthermore, the reporting quality of IPTW analysis was uneven when handling multi-classification treatment, especially regarding assumptions and model construction. This may affect our accurate assessment of the benefit from ACT in patients with stage T4. Here, in the T4 stage, as demonstrated by the landmark analysis, patients could archive long-term survival benefits from the ACT. The landmark analysis, as proposed by Anderson et al., entails the selection of a specific time point during the follow-up (considered as a landmark) and then assessing patient characteristics at that designated landmark timepoint (37). However, in the T3 stage, EOCRC patients could not yield benefit from the ACT after the adjustments of confounders.

Intriguingly, the median number of rLNs in the two EOCRC cohorts exceeded 16, substantially surpassing the recommended threshold of 12 typically advised for the broader CRC patient population (38). This could be explained by that more spread of tumor resection and colon tissues among the EOCRC patients due to the advanced T stage and poorly differentiated tumor grade. The distribution of them was similar between ACT and non-ACT groups before the PSM, suggesting no significantly prognostic effect of them. Herein, we did not divide EOCRC patients into corresponding subgroups. Besides, patients with EOCRC often suffer from ACT-induced nausea and vomiting, particularly women with low BMI and they should be allocated with enhanced prophylactic use of antiemetic drugs (39–41). Therefore, we should avoid intensive and inefficient treatments for II stage young patients, and in our study, we recommend that T4 stage patients should adopt the postoperative ACT actively.

Our findings on the limited benefit of ACT in stage II EOCRC patients, with notable exceptions in T4 stage, align with recent studies on the management of EOCRC and stage II colon cancer. Tang et al. identified prognostic factors for EOCRC patients post-chemotherapy using the SEER database and developed a Nomogram model for survival prediction (42). Their work underscores the complexity of prognostic analysis in EOCRC, while our study directly addresses the therapeutic necessity of ACT in stage II EOCRC patients, identifying T4 stage as a potential subgroup benefiting from ACT. This complements Tang et al.’s work by providing specific insights into treatment efficacy in different stages of EOCRC.

Varghese reviewed the role of adjuvant chemotherapy in stage II colon cancer, highlighting the heterogeneity of this patient group and the ongoing controversy regarding ACT (43). Our study builds on this by focusing on the EOCRC population, which has distinct clinical and molecular features. We found that dMMR/pMMR status does not predict ACT efficacy in stage II EOCRC patients, echoing Varghese’s discussion on the limitations of current biomarkers. Our findings on the potential long-term benefits of ACT in T4 stage patients offer a nuanced perspective on the role of ACT in high-risk subgroups, contributing to the ongoing debate on its use in stage II colon cancer.

This study adds to the existing literature by providing a detailed analysis of ACT therapeutic necessity in stage II EOCRC patients, particularly highlighting the potential benefits in T4 stage patients. This work complements recent studies by offering specific insights into the treatment of EOCRC, a population with unique clinical needs and challenges.

### Limitations

Some limitations of the study should be mentioned. Firstly, this observational study based on two retrospective cohorts, inherently had selective bias and confounding factors. Here, we reduced them as efficiently as possible for the measured confounders with the PSM method and multivariate Cox analyses. Secondly, given that the proportion of T4 stage in our cohort was low and MMR status was unavailable in the SEER cohort, we conducted subgroup analyses solely within the available cohort. Moreover, considering that numerous studies have demonstrated that dMMR patients benefit from immunotherapy but not from chemotherapy alone, and that no OS differences exist between dMMR and pMMR patients treated with chemotherapy alone, we did not perform subgroup analyses based on MMR status. Instead, we focused on T stage as a critical factor for treatment decision-making, aiming to explore the necessity of adjuvant chemotherapy in stage II EOCRC patients. Thirdly, there could still be other factors affecting clinical prognosis of EOCRC, including tumor mutation burden, such as *TP53* and *CTNNB1 (* 44, 45). Lastly, in our study, it was initially demonstrated that MMR status may not serve as an effective predictor for determining the necessity of ACT in patients with EOCRC. However, given the limitations of our sample size and study design, these findings require further validation through large-scale, prospective studies or randomized controlled trials to establish the robustness and generalizability of this conclusion. It is important to note that our sample size was still relatively small, which may limit the generalizability and robustness of our findings. Indeed, very few high-quality indicators are perfect, and the aim of our study was to explore the efficacy of postoperative ACT on the EOCRC with II stage, which could provide powerful evidence for the avoiding the overtreatment for EOCRC patients.

## Conclusions

Among stage II EOCRC patients, most may derive limited prognostic benefits from postoperative ACT. However, those at T4 stage might observe survival benefits three years post-surgery. Furthermore, MMR status may not serve as an effective predictor for ACT adoption following surgery in these stage II EOCRC patients. Our study highlights the importance of avoiding unnecessary ACT for stage II EOCRC patients, especially those with T3 stage, and suggests that T4 stage patients should actively consider postoperative ACT. Future research should focus on identifying more effective biomarkers to guide treatment decisions and validate our findings in larger, prospective studies.

## Data Availability

The original contributions presented in the study are included in the article/**Supplementary Material**. Further inquiries can be directed to the corresponding authors.
