# Supplementary material for: Postoperative adjuvant chemotherapy in patients with stage II early onset colorectal cancer: exploration and discovery using real-world data and the SEER database
Source: Front Oncol. 2025 May 30;15:1566569. doi: 10.3389/fonc.2025.1566569 (PMC12162292; doi:10.3389/fonc.2025.1566569)
Supplement: Supplementary file 1 [file DataSheet1.docx]

**Supplementary Online Content**

**Postoperative Adjuvant Chemotherapy in Patients with Stage II Early Onset Colorectal Cancer: Exploration and Discovery**

Table1. Basic characteristics of the II stage EOCRC before and after PSM in the XJCRC cohort

Table2. Basic characteristics of the II stage EOCRC before and after PSM in the SEER cohort

Table3. Basic characteristics of dMMR EOCRC before and after PSM in the XJCRC cohort

Table4. Basic characteristics of pMMR EOCRC before and after PSM in the XJCRC cohort

Table5. Basic characteristics of the T3 stage EOCRC before and after PSM in the SEER cohort

Table6. Basic characteristics of the T4 stage EOCRC before and after PSM in the SEER cohort

This supplementary material has been provided by the authors to give readers additional information about their work.

**Table S1 Basic characteristics of the II stage EOCRC before and after PSM in the XJCRC cohort**

| Characteristics | Before PSM | | P value | After PSM | | P value |
| --- | --- | --- | --- | --- | --- | --- |
|  | Non-ACT | ACT |  | Non-ACT | ACT |  |
|  | N=296 | N=50 |  | N=49 | N=49 |  |
| Gender: |  |  | 0.509 |  |  | 0.838 |
| female | 118 (39.9%) | 23 (46.0%) |  | 20 (40.8%) | 22 (44.9%) |  |
| male | 178 (60.1%) | 27 (54.0%) |  | 29 (59.2%) | 27 (55.1%) |  |
| Age | 43.5 [38.0;47.0] | 43.0 [37.0;47.0] | 0.574 | 42.0 [39.0;47.0] | 43.0 [37.0;47.0] | 0.781 |
| Height (cm) | 169 [162;173] | 169 [162;172] | 0.99 | 167 (7.41) | 168 (7.25) | 0.304 |
| Weight (Kg) | 63.0 [54.0;72.0] | 64.8 [55.0;75.0] | 0.751 | 63.4 (13.0) | 65.2 (14.1) | 0.509 |
| Albumin (g/L) | 43.1 [38.2;45.9] | 43.0 [39.1;45.0] | 0.732 | 43.5 [39.7;46.1] | 42.9 [39.1;45.0] | 0.536 |
| CEA (ng/mL) | 2.48 [1.35;5.79] | 1.99 [1.08;3.96] | 0.125 | 2.19 [1.31;6.40] | 1.99 [1.08;3.58] | 0.269 |
| CA199 (U/mL) | 10.7 [5.90;18.5] | 9.54 [6.64;20.2] | 0.88 | 12.1 [6.19;29.3] | 9.45 [6.58;20.3] | 0.597 |
| CA125 (U/mL) | 12.6 [8.86;18.4] | 12.9 [8.30;20.6] | 0.822 | 12.4 [9.39;17.5] | 13.0 [8.30;20.7] | 0.859 |
| rLNs | 19.0 [16.0;23.0] | 17.0 [9.00;20.0] | 0.005 | 19.0 [16.0;22.0] | 17.0 [8.00;20.0] | 0.094 |
| S100: |  |  | 0.344 |  |  | 0.785 |
| negative | 234 (79.1%) | 43 (86.0%) |  | 40 (81.6%) | 42 (85.7%) |  |
| positive | 62 (20.9%) | 7 (14.0%) |  | 9 (18.4%) | 7 (14.3%) |  |
| CD34: |  |  | 0.666 |  |  | 0.643 |
| negative | 225 (76.0%) | 36 (72.0%) |  | 38 (77.6%) | 35 (71.4%) |  |
| positive | 71 (24.0%) | 14 (28.0%) |  | 11 (22.4%) | 14 (28.6%) |  |
| D240: |  |  | 0.398 |  |  | 0.468 |
| negative | 223 (75.3%) | 41 (82.0%) |  | 36 (73.5%) | 40 (81.6%) |  |
| positive | 73 (24.7%) | 9 (18.0%) |  | 13 (26.5%) | 9 (18.4%) |  |
| BMI (kg/m2) | 22.8 [20.2;25.2] | 22.5 [19.9;24.7] | 0.319 | 21.5 [19.6;23.8] | 22.6 [19.9;24.8] | 0.346 |
| T stage: |  |  | 0.896 |  |  | 0.773 |
| T3 | 255 (86.1%) | 44 (88.0%) |  | 41 (83.7%) | 43 (87.8%) |  |
| T4 | 41 (13.9%) | 6 (12.0%) |  | 8 (16.3%) | 6 (12.2%) |  |
| MMR status: |  |  | 0.605 |  |  | 0.651 |
| dMMR | 85 (28.7%) | 12 (24.0%) |  | 15 (30.6%) | 12 (24.5%) |  |
| pMMR | 211 (71.3%) | 38 (76.0%) |  | 34 (69.4%) | 37 (75.5%) |  |

ACT: adjuvant chemotherapy; PSM: Propensity Score Matching; EOCRC: early onset colorectal cancer; rLNs: retrieved lymph nodes; XJCRC: Xijing hospital CRC.

**Table S2 Basic characteristics of the II stage EOCRC before and after PSM in the SEER cohort**

| Characteristics | Before PSM | | P value | After PSM | | P value |
| --- | --- | --- | --- | --- | --- | --- |
|  | Non-ACT | ACT |  | Non-ACT | ACT |  |
|  | N=2067 | N=1163 |  | N=1000 | N=1000 |  |
| Age | 45.0 [40.0;47.0] | 44.0 [39.0;47.0] | 0.0195 | 44.0 [39.0;47.0] | 44.0 [39.0;47.0] | 0.601 |
| Gender: |  |  | 0.019 |  |  | 0.964 |
| female | 946 (45.8%) | 583 (50.1%) |  | 507 (50.7%) | 505 (50.5%) |  |
| male | 1121 (54.2%) | 580 (49.9%) |  | 493 (49.3%) | 495 (49.5%) |  |
| Race: |  |  | 0.219 |  |  | 0.149 |
| white | 1530 (74.0%) | 865 (74.4%) |  | 732 (73.2%) | 750 (75.0%) |  |
| black | 290 (14.0%) | 179 (15.4%) |  | 148 (14.8%) | 153 (15.3%) |  |
| other | 228 (11.0%) | 114 (9.8%) |  | 110 (11.0%) | 94 (9.4%) |  |
| unknown | 19 (1.0%) | 5 (0.4%) |  | 10 (1.0%) | 3 (0.3%) |  |
| Marriage: |  |  | 0.116 |  |  | 0.717 |
| married | 1131 (54.7%) | 647 (55.6%) |  | 552 (55.2%) | 557 (55.7%) |  |
| unmarried | 789 (38.2%) | 455 (39.1%) |  | 398 (39.8%) | 386 (38.6%) |  |
| Unknown | 147 (7.11%) | 61 (5.3%) |  | 50 (5.0%) | 57 (5.7%) |  |
| Grade: |  |  | <0.001 |  |  | 1 |
| well differentiated | 193 (9.3%) | 68 (5.9%) |  | 65 (6.5%) | 66 (6.6%) |  |
| moderately differentiated | 1527 (73.9%) | 835 (71.8%) |  | 736 (73.6%) | 733 (73.3%) |  |
| poorly differentiated | 217 (10.5%) | 188 (16.2%) |  | 140 (14.0%) | 143 (14.3%) |  |
| undifferentiated | 60 (2.9%) | 46 (3.9%) |  | 35 (3.5%) | 34 (3.4%) |  |
| unknown | 70 (3.4%) | 26 (2.2%) |  | 24 (2.4%) | 24 (2.4%) |  |
| T stage: |  |  | <0.001 |  |  | 0.774 |
| T3 | 1856 (89.8%) | 809 (69.5%) |  | 805 (80.5%) | 802 (80.2%) |  |
| T4 | 211 (10.2%) | 354 (30.5%) |  | 195 (19.5%) | 198 (19.8%) |  |
| Tumor size | 55.0 [40.0;72.0] | 55.0 [40.0;80.0] | 0.002 | 53.5 [40.0;74.2] | 55.0 [40.0;75.0] | 0.128 |
| rLNs | 21.0 [16.0;30.0] | 21.0 [15.0;29.0] | 0.031 | 20.0 [15.0;28.0] | 20.0 [15.0;29.0] | 0.777 |
| ACT: adjuvant chemotherapy; PSM: Propensity Score Matching; SEER: Surveillance, Epidemiology, and End Results; rLNs: retrieved lymph nodes. | | | | | | |

**Table S3 Basic characteristics of dMMR EOCRC before and after PSM in the XJCRC data cohort**

| Characteristics | Before PSM | | P value | After PSM | | P value |
| --- | --- | --- | --- | --- | --- | --- |
|  | Non-ACT | ACT |  | Non-ACT | ACT |  |
|  | N=85 | N=12 |  | N=11 | N=11 |  |
| Gender: |  |  | 0.214 |  |  | 0.669 |
| female | 32 (37.6%) | 7 (58.3%) |  | 5 (45.5%) | 7 (63.6%) |  |
| male | 53 (62.4%) | 5 (41.7%) |  | 6 (54.5%) | 4 (36.4%) |  |
| Age | 42.0 [35.0;46.0] | 44.0 [40.8;46.2] | 0.326 | 45.0 [38.5;46.0] | 44.0 [40.5;45.5] | 0.62 |
| Height (cm) | 168 (7.65) | 166 (6.11) | 0.311 | 168 (5.95) | 165 (6.25) | 0.357 |
| Weight (Kg) | 62.5 [54.0;72.0] | 55.0 [52.5;59.5] | 0.052 | 58.7 (13.5) | 56.5 (6.56) | 0.633 |
| Albumin (g/L) | 41.3 [35.6;44.9] | 41.2 [33.6;43.4] | 0.539 | 40.2 (6.49) | 39.0 (6.61) | 0.665 |
| CEA (ng/mL) | 1.92 [1.11;3.50] | 1.53 [0.81;3.10] | 0.288 | 1.60 [1.28;1.92] | 1.60 [0.92;3.26] | 0.921 |
| CA199 (U/mL) | 8.32 [5.06;18.9] | 9.81 [7.57;22.1] | 0.268 | 6.94 [5.88;8.07] | 10.2 [8.13;23.9] | 0.02 |
| CA125 (U/mL) | 14.0 [9.51;23.7] | 16.0 [10.6;20.7] | 0.952 | 20.2 (9.73) | 16.8 (6.77) | 0.357 |
| rLNs | 19.0 [16.0;24.0] | 18.5 [12.8;23.0] | 0.506 | 19.0 [18.0;23.0] | 18.0 [8.50;23.0] | 0.551 |
| S100: |  |  | 1 |  |  | 1 |
| negative | 62 (72.9%) | 9 (75.0%) |  | 9 (81.8%) | 8 (72.7%) |  |
| positive | 23 (27.1%) | 3 (25.0%) |  | 2 (18.2%) | 3 (27.3%) |  |
| CD34: |  |  | 1 |  |  | 1 |
| negative | 64 (75.3%) | 9 (75.0%) |  | 9 (81.8%) | 8 (72.7%) |  |
| positive | 21 (24.7%) | 3 (25.0%) |  | 2 (18.2%) | 3 (27.3%) |  |
| D240: |  |  | 0.724 |  |  | 1 |
| negative | 63 (74.1%) | 10 (83.3%) |  | 8 (72.7%) | 9 (81.8%) |  |
| positive | 22 (25.9%) | 2 (16.7%) |  | 3 (27.3%) | 2 (18.2%) |  |
| BMI (kg/m2) | 23.0 [20.0;25.6] | 20.4 [19.7;22.2] | 0.072 | 20.4 [19.2;24.7] | 20.6 [19.9;22.4] | 0.922 |
| T stage: |  |  | 0.203 |  |  | 1 |
| T3 | 70 (82.4%) | 12 (100%) |  | 11 (100%) | 11 (100%) |  |
| T4 | 15 (17.6%) | 0 (0.00%) |  |  |  |  |

ACT: adjuvant chemotherapy; PSM: Propensity Score Matching; rLNs: retrieved lymph nodes; XJCRC: Xijing hospital CRC.

**Table S4 Basic characteristics of pMMR EOCRC before and after PSM in the XJCRC cohort**

| Characteristics | Before PSM | | P value | After PSM | | P value |
| --- | --- | --- | --- | --- | --- | --- |
|  | Non-ACT | ACT |  | Non-ACT | ACT |  |
|  | N=211 | N=38 |  | N=34 | N=34 |  |
| Gender: |  |  | 1 |  |  | 0.464 |
| female | 86 (40.8%) | 16 (42.1%) |  | 17 (50.0%) | 13 (38.2%) |  |
| male | 125 (59.2%) | 22 (57.9%) |  | 17 (50.0%) | 21 (61.8%) |  |
| Age | 44.0 [39.0;47.0] | 42.5 [35.2;47.0] | 0.227 | 46.0 [40.0;47.8] | 44.0 [37.0;47.8] | 0.257 |
| Height (cm) | 169 [162;172] | 170 [164;173] | 0.454 | 168 (8.55) | 169 (6.25) | 0.834 |
| Weight (Kg) | 64.0 [55.0;72.0] | 67.0 [56.0;75.0] | 0.423 | 61.7 (12.9) | 65.9 (14.3) | 0.233 |
| Albumin (g/L) | 43.7 [39.5;46.1] | 43.9 [39.2;45.4] | 0.867 | 42.0 [39.4;45.6] | 43.5 [39.2;45.0] | 0.844 |
| CEA (ng/mL) | 3.05 [1.40;6.34] | 2.18 [1.10;4.20] | 0.191 | 2.67 [1.68;5.00] | 1.99 [1.08;3.30] | 0.038 |
| CA199 (U/mL) | 12.1 [6.46;18.5] | 9.44 [5.73;19.7] | 0.653 | 11.5 [5.56;18.2] | 8.61 [5.43;19.2] | 0.792 |
| CA125 (U/mL) | 12.1 [8.05;17.6] | 12.1 [7.91;20.5] | 0.783 | 13.6 [7.96;19.5] | 12.1 [7.91;21.1] | 0.589 |
| rLNs | 18.0 [16.0;22.0] | 16.0 [9.00;19.8] | 0.005 | 18.0 [17.0;21.8] | 16.5 [11.00;19.0] | 0.11 |
| S100: |  |  | 0.336 |  |  | 0.732 |
| negative | 39 (18.5%) | 4 (10.5%) |  | 6 (17.6%) | 4 (11.8%) |  |
| positive | 172 (81.5%) | 34 (89.5%) |  | 28 (82.4%) | 30 (88.2%) |  |
| CD34: |  |  | 0.626 |  |  | 1 |
| negative | 161 (76.3%) | 27 (71.1%) |  | 26 (76.5%) | 25 (73.5%) |  |
| positive | 50 (23.7%) | 11 (28.9%) |  | 8 (23.5%) | 9 (26.5%) |  |
| D240: |  |  | 0.573 |  |  | 0.75 |
| negative | 160 (75.8%) | 31 (81.6%) |  | 27 (79.4%) | 29 (85.3%) |  |
| positive | 51 (24.2%) | 7 (18.4%) |  | 7 (20.6%) | 5 (14.7%) |  |
| BMI (kg/m2) | 22.8 [20.5;25.1] | 22.7 [20.0;25.4] | 0.922 | 21.6 (3.37) | 22.9 (3.92) | 0.142 |
| T stage: |  |  | 0.598 |  |  | 1 |
| T3 | 185 (87.7%) | 32 (84.2%) |  | 29 (85.3%) | 29 (85.3%) |  |
| T4 | 26 (12.3%) | 6 (15.8%) |  | 5 (14.7%) | 5 (14.7%) |  |

ACT: adjuvant chemotherapy; PSM: Propensity Score Matching; rLNs: retrieved lymph nodes; XJCRC: Xijing hospital CRC.

**Table S5 Basic characteristics of the T3 stage EOCRC before and after PSM in the SEER cohort**

| Characteristics | Before PSM | | P value | After PSM | | P value |
| --- | --- | --- | --- | --- | --- | --- |
|  | Non-ACT | ACT |  | Non-ACT | ACT |  |
|  | N=1856 | N=809 |  | N=178 | N=178 |  |
| Age | 45.0 [40.0;47.0] | 44.0 [39.0;47.0] | 0.002 | 44.0 [39.0;47.0] | 43.5 [39.0;47.0] | 0.568 |
| Gender: |  |  | 0.008 |  |  | 0.915 |
| female | 859 (46.3%) | 420 (51.9%) |  | 78 (43.8%) | 76 (42.7%) |  |
| male | 997 (53.7%) | 389 (48.1%) |  | 100 (56.2%) | 102 (57.3%) |  |
| Race: |  |  | 0.174 |  |  | 0.386 |
| white | 1366 (73.6%) | 608 (75.2%) |  | 139 (78.1%) | 133 (74.7%) |  |
| black | 261 (14.1%) | 122 (15.1%) |  | 24 (13.5%) | 26 (14.6%) |  |
| other | 212 (11.4%) | 76 (9.39%) |  | 13 (7.30%) | 19 (10.7%) |  |
| unknown | 17 (0.92%) | 3 (0.37%) |  | 2 (1.12%) | 0 (0.00%) |  |
| Marriage: |  |  | 0.292 |  |  | 0.863 |
| married | 1028 (55.4%) | 447 (55.3%) |  | 94 (52.8%) | 90 (50.6%) |  |
| unmarried | 132 (7.11%) | 45 (5.56%) |  | 10 (5.62%) | 12 (6.74%) |  |
| Unknown | 696 (37.5%) | 317 (39.2%) |  | 74 (41.6%) | 76 (42.7%) |  |
| Grade: |  |  | <0.001 |  |  | 0.618 |
| well differentiated | 164 (8.84%) | 42 (5.19%) |  | 18 (10.1%) | 22 (12.4%) |  |
| moderately differentiated | 1389 (74.8%) | 608 (75.2%) |  | 122 (68.5%) | 115 (64.6%) |  |
| poorly differentiated | 195 (10.5%) | 119 (14.7%) |  | 20 (11.2%) | 27 (15.2%) |  |
| undifferentiated | 52 (2.80%) | 25 (3.09%) |  | 8 (4.49%) | 8 (4.49%) |  |
| unknown | 56 (3.02%) | 15 (1.85%) |  | 10 (5.62%) | 6 (3.37%) |  |
| Tumor size | 52.0 [40.0;70.0] | 54.0 [40.0;74.0] | 0.398 | 65.0 [50.0;89.5] | 65.0 [45.0;93.5] | 0.884 |
| rLNs | 21.0 [16.0;30.0] | 20.0 [15.0;28.0] | 0.001 | 20.0 [15.0;28.0] | 20.0 [15.0;30.0] | 0.922 |
| ACT: adjuvant chemotherapy; PSM: Propensity Score Matching; rLNs: retrieved lymph nodes. | | | | | | |

**Table S6 Basic characteristics of the T4 stage EOCRC before and after PSM in the SEER cohort**

| Characteristics | Before PSM | | P value | After PSM | | P value |
| --- | --- | --- | --- | --- | --- | --- |
|  | Non-ACT | ACT |  | Non-ACT | ACT |  |
|  | N=211 | N=354 |  | N=178 | N=178 |  |
| Age | 44.0 [39.0;47.0] | 44.0 [38.0;47.0] | 0.189 | 44.0 [39.0;47.0] | 43.5 [39.0;47.0] | 0.568 |
| Gender: |  |  | 0.305 |  |  | 0.915 |
| female | 87 (41.2%) | 163 (46.0%) |  | 78 (43.8%) | 76 (42.7%) |  |
| male | 124 (58.8%) | 191 (54.0%) |  | 100 (56.2%) | 102 (57.3%) |  |
| Race: |  |  | 0.45 |  |  | 0.386 |
| white | 164 (77.7%) | 257 (72.6%) |  | 139 (78.1%) | 133 (74.7%) |  |
| black | 29 (13.7%) | 57 (16.1%) |  | 24 (13.5%) | 26 (14.6%) |  |
| other | 16 (7.6%) | 38 (10.7%) |  | 13 (7.3%) | 19 (10.7%) |  |
| unknown | 2 (1.0%) | 2 (0.6%) |  | 2 (1.1%) | 0 (0.0%) |  |
| Marriage: |  |  | 0.142 |  |  | 0.863 |
| married | 103 (48.8%) | 200 (56.5%) |  | 94 (52.8%) | 90 (50.6%) |  |
| unmarried | 15 (7.1%) | 16 (4.5%) |  | 10 (5.6%) | 12 (6.7%) |  |
| Unknown | 93 (44.1%) | 138 (39.0%) |  | 74 (41.6%) | 76 (42.7%) |  |
| Grade: |  |  | 0.002 |  |  | 0.618 |
| well differentiated | 29 (13.7%) | 26 (7.4%) |  | 18 (10.1%) | 22 (12.4%) |  |
| moderately differentiated | 138 (65.4%) | 227 (64.1%) |  | 122 (68.5%) | 115 (64.6%) |  |
| poorly differentiated | 22 (10.4%) | 69 (19.5%) |  | 20 (11.2%) | 27 (15.2%) |  |
| undifferentiated | 8 (3.8%) | 21 (5.9%) |  | 8 (4.5%) | 8 (4.5%) |  |
| unknown | 14 (6.7%) | 11 (3.1%) |  | 10 (5.7%) | 6 (3.3%) |  |
| Tumor size | 65.0 [48.0;87.5] | 70.0 [45.0;90.0] | 0.73 | 65.0 [50.0;89.5] | 65.0 [45.0;93.5] | 0.884 |
| rLNs | 19.0 [14.0;26.5] | 22.0 [16.0;31.0] | 0.028 | 20.0 [15.0;28.0] | 20.0 [15.0;30.0] | 0.922 |
| ACT: adjuvant chemotherapy; PSM: Propensity Score Matching; rLNs: retrieved lymph nodes; XJCRC: Xijing hospital CRC. | | | | | | |


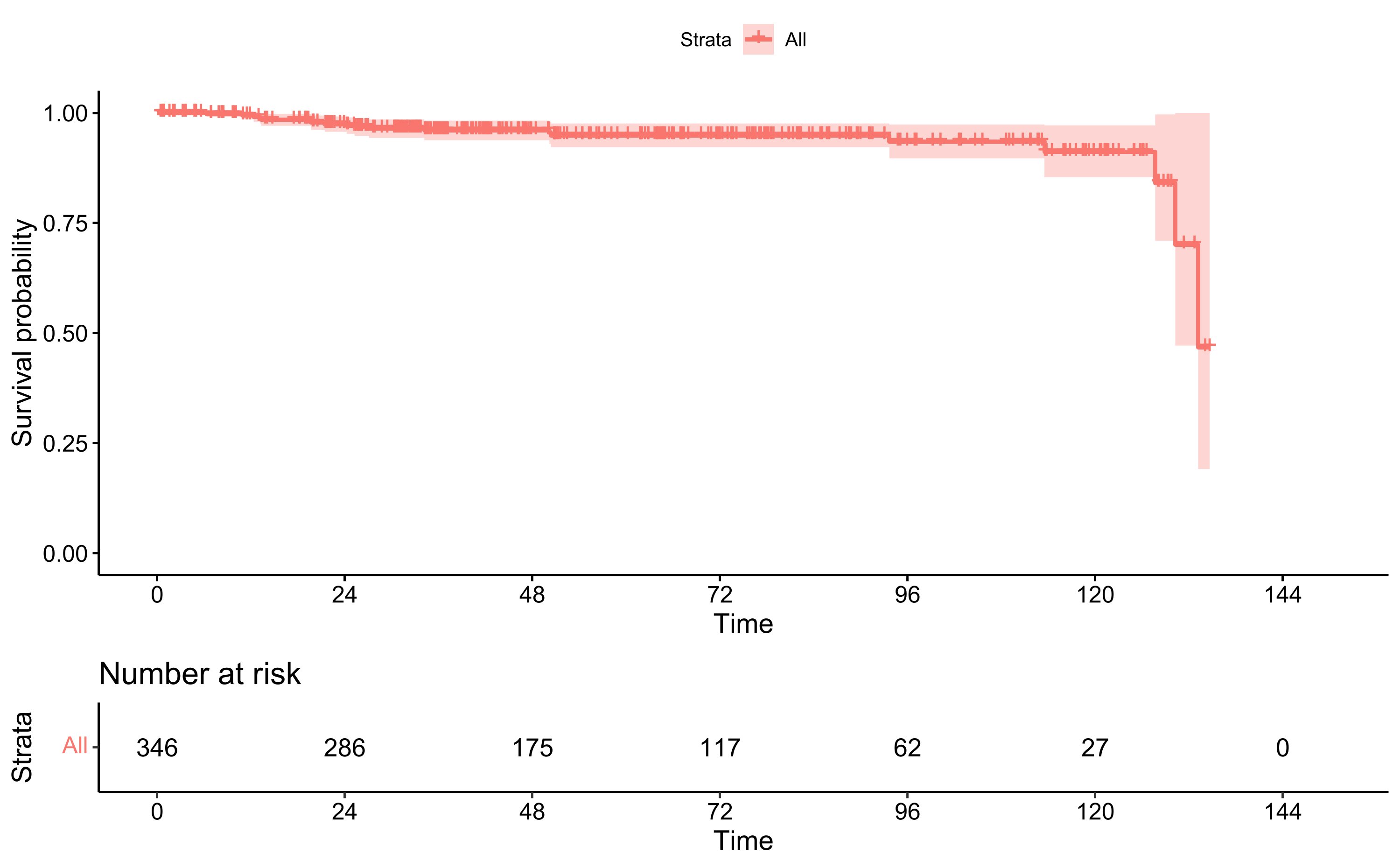


Supplementary Figure 1: Ka plan-Meier survival curves for Stage II EOCRC patients.


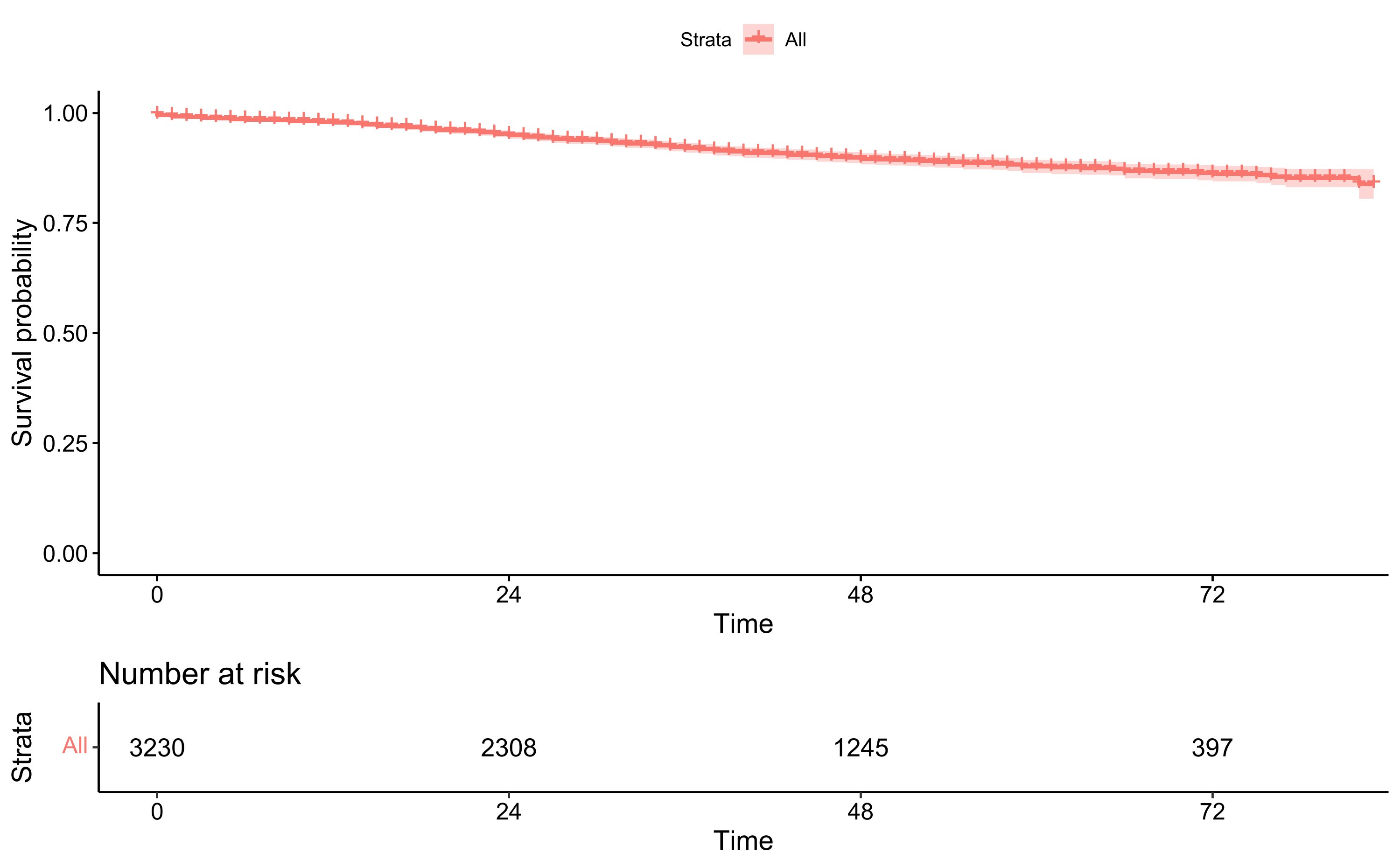


Supplementary Figure 2: Kaplan-Meier survival curves for Stage II EOCRC patients in the SEER cohort.


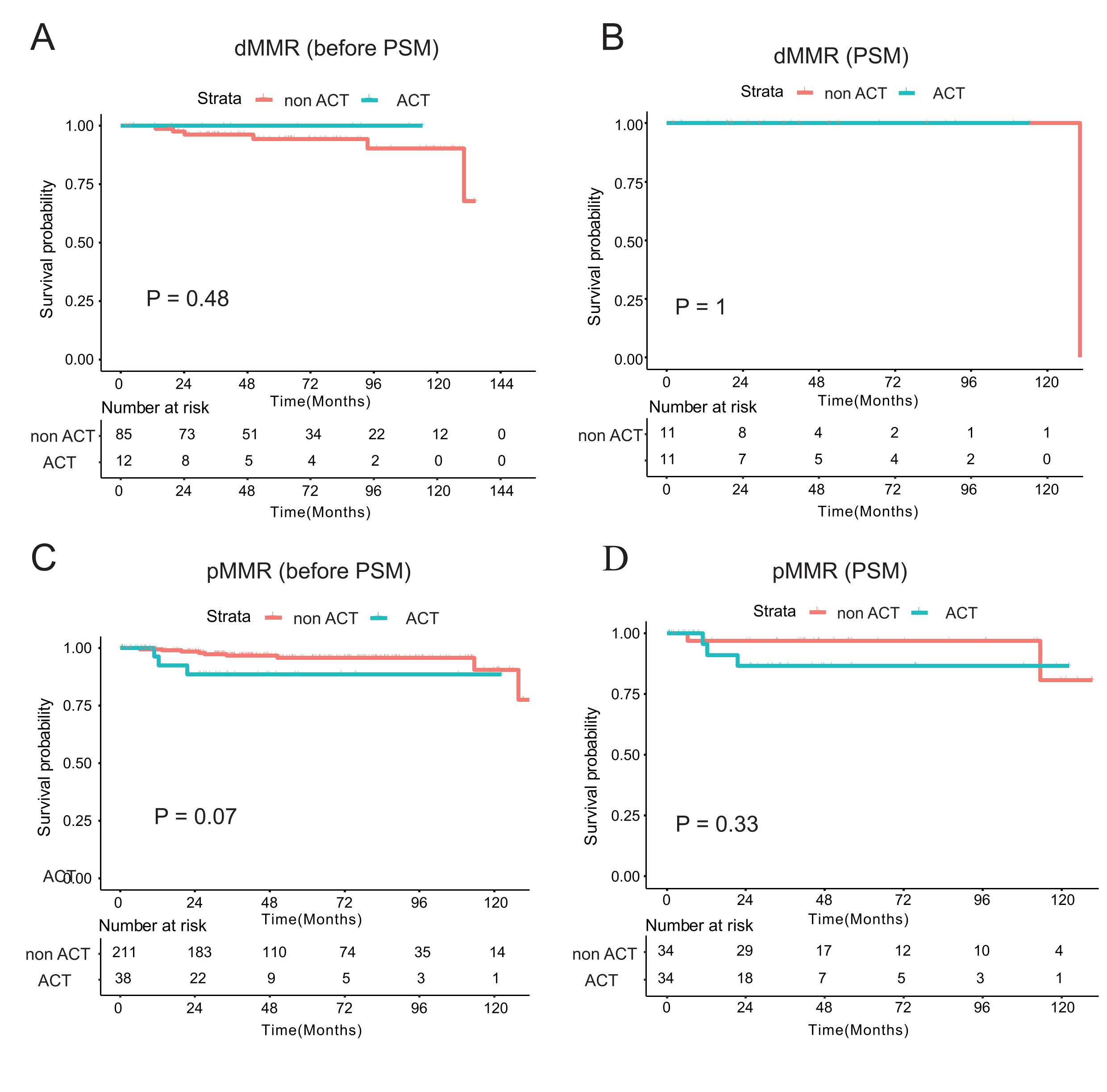


Supplementary Figure 3: Kaplan-Meier survival analysis of Stage II EOCRC patients by treatment status. (A) Before PSM analysis of non-ACT and ACT patients with dMMR. (B) Survival curves of non-ACT and ACT patients with dMMR after PSM. (C)Before PSM analysis of non-ACT and ACT patients with pMMR. (D) Survival curves of non-ACT and ACT patients with PMMR after PSM.
